# Pre-hospital treatment duration and efficacy of remote ischaemic conditioning in the RESIST randomised-controlled trial

**DOI:** 10.1093/esj/aakaf015

**Published:** 2026-01-01

**Authors:** Aravind Ganesh, David Gaist, Boris Modrau, Martin Faurholdt Gude, Anne Brink Behrndtz, Grethe Andersen, Claus Ziegler Simonsen, Rolf Ankerlund Blauenfeldt

**Affiliations:** Calgary Stroke Program, Departments of Clinical Neurosciences, Community Health Sciences, and Radiology, the Hotchkiss Brain Institute, and the O’Brien Institute for Public Health, University of Calgary Cumming School of Medicine, Calgary, Canada; Research Unit for Neurology, Odense University Hospital, University of Southern Denmark, Odense, Denmark; Department of Neurology, Aalborg University Hospital, Aalborg, Denmark; Department of Research and Development, Prehospital Emergency Medical Services, Central Denmark Region, Aarhus, Denmark; Department of Neurology, Regional Hospital Gødstrup, Gødstrup, Denmark; Department of Neurology, Aarhus University Hospital, Aarhus, Denmark; Department of Clinical Medicine, Aarhus University, Aarhus, Denmark; Department of Neurology, Aarhus University Hospital, Aarhus, Denmark; Department of Clinical Medicine, Aarhus University, Aarhus, Denmark; Department of Neurology, Aarhus University Hospital, Aarhus, Denmark; Department of Clinical Medicine, Aarhus University, Aarhus, Denmark

**Keywords:** remote ischaemic conditioning, ischaemic stroke, intracerebral haemorrhage, stroke, outcomes

## Abstract

**Introduction:**

Remote ischaemic conditioning (RIC) initiated pre-hospital did not improve 90-day functional outcomes after acute stroke in the RESIST trial. The duration of treatment pre-reperfusion modifies treatment effect for other neuroprotective therapies. We examined whether the effects of RIC might be modified by the duration of pre-hospital treatment.

**Patients and methods:**

This post-hoc analysis of the RESIST randomised-controlled trial (ClinicalTrials.gov: NCT03481777) included patients who presented with pre-hospital stroke symptoms < 4 hours, randomised to RIC or sham, diagnosed with acute ischaemic stroke (AIS) or ICH (modified intention-to-treat [mITT] cohort). Patients were stratified by time from randomisation to hospital admission (ie, pre-hospital treatment duration). The primary outcome was shift in 90-day mRS; secondary outcomes were 90-day mRS 0–2 and 24-hour neurological improvement (NIHSS).

**Results:**

Among 902 mITT patients (AIS, *n* = 737; ICH, *n* = 165), median randomisation-to-admission time was 29.4 minutes (IQR: 19.6–39.4) and median onset-to-admission time was 88 minutes (IQR: 62.4–131.3). Across pre-hospital treatment duration strata, RIC conferred no significant benefit on 90-day mRS, mRS 0–2 or early NIHSS improvement in the combined, AIS or ICH populations. In patients with AIS receiving reperfusion therapy, stratification by transport time likewise revealed no efficacy differences. No significant interaction was observed between RIC and pre-hospital treatment duration for any outcome.

**Conclusion:**

Longer pre-hospital treatment duration was not associated with efficacy of RIC in the RESIST trial including in patients with AIS who received reperfusion therapies. Findings may not apply to settings where RIC could be routinely administered for longer periods. We found no treatment duration-dependent benefit of pre-hospital RIC, at least when durations are under an hour.

## Graphical Abstract

**Graphical abstract:**
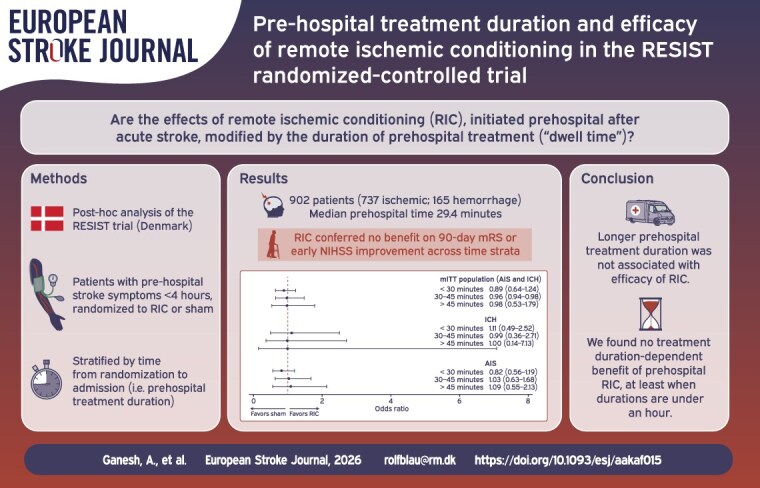

## Introduction

The pre-hospital setting of acute stroke care remains a challenging frontier. Many patients experience pre-hospital delays in arrival to stroke centres, and in many countries, patients often require an hour or more to be transported to hospital even under the best of circumstances due to geographic realities.^1^^,^^2^ Unfortunately, there are no approved pre-hospital treatments for stroke that can be administered in conventional ambulances, meaning patients’ outcomes are fully dependent on how much brain tissue can be salvaged in hospital.^3^

Remote ischaemic conditioning (RIC) is a promising strategy to mitigate infarct growth in acute ischaemic stroke (AIS) as well as haematoma expansion or perihaematomal edema in ICH, offering a potential pre-hospital treatment strategy for acute stroke, regardless of whether ischaemic or haemorrhagic.^4^^,^^5^ Remote ischaemic conditioning induces brief periods of ischaemia–reperfusion in a limb to protect a remote organ (eg, brain) from injury through humoral and neuronal-mediated responses promoting cell survival/repair and inhibiting apoptosis/inflammation.^6^^,^^7^ In the pre-hospital setting, a Danish proof-of-concept trial administered RIC in the ambulance to patients with suspected stroke, demonstrating the safety of RIC even in patients with ICH.^8^ There were more TIAs and less severe strokes in the RIC group, and ambulance transport was relatively short, interfering with the application of RIC, but after adjustment for baseline perfusion and diffusion lesion severity, post-hoc voxel-wise analyses suggested that RIC lowered the tissue risk of infarction. This finding was followed up by the Remote Ischemic Conditioning in Patients With Acute Stroke Trial (RESIST) trial, a randomised clinical trial that included 1500 patients with a suspected stroke in the pre-hospital setting.^9^ The RESIST trial found that RIC treatment started in the pre-hospital setting, compared with sham, did not significantly improve functional outcomes according to the mRS at 90 days in patients with a confirmed diagnosis of stroke (odds ratio [OR] for a favorable shift on the mRS score: 0.95).^9^

Recently, other trials of potential pre-hospital therapies have identified the duration of treatment received prior to reperfusion as a key treatment effect modifier. For instance, in the Field Randomization of Nerinetide Therapy in Early Responders (FRONTIER) trial, pre-hospital nerinetide did not improve neurological functional outcomes in 532 patients with suspected ischaemic stroke, though it appeared to benefit patients with AIS who were selected for reperfusion therapies within 3 hours of symptom onset.^10^ Subsequently, a post-hoc meta-analysis of individual patient data from 3 trials of nerinetide,^10–12^ which was restricted to patients with AIS enrolled within 3 hours and selected for reperfusion without previous thrombolysis, suggested that a longer interval between the administration of nerinetide and initiation of reperfusion (termed “dwell time”) seemed to modify the effect of nerinetide, with longer times providing greater clinical benefit.^13^ This idea may also apply to RIC since the endogenous pathways activated by RIC do appear to be time-dependent. For example, prior basic or translational science studies have indicated that the magnitude of the protective mechanisms conferred with early phase RIC rises over the course of several hours following the onset of treatment, thought to be caused by rapid posttranslational modification of pre-existing proteins.^14^ It is conceivable that this process might be further enhanced by greater duration of exposure to RIC prior to the initiation of in-hospital stroke care, translating into greater clinical benefit. Therefore, we examined whether the effects of RIC in the RESIST trial might be modified by the duration of pre-hospital treatment that patients received, including among patients with AIS who did or did not receive reperfusion therapies.

**Table 1:** Baseline characteristics in patients enrolled in the RESIST trial, grouped by pre-hospital treatment duration or randomisation-to-admission time

**Characteristic**	**Randomisation-to-admission time (pre-hospital treatment duration)**			***P*-value**
	**<30 min**	**30–45 min**	**>45 min**	
***n***	459	298	145	
**Age, median (IQR)**	72 (62, 80)	72 (61, 79)	75 (66, 79)	.18
**Sex, *n* (%)**				
Male	275 (59.9%)	195 (65.4%)	97 (66.9%)	.17
Female	184 (40.1%)	103 (34.6%)	48 (33.1%)	
**Comorbidities, *n* (%)**				
Hypertension	274 (59.7%)	191 (64.1%)	102 (70.3%)	.060
Diabetes	57 (12.4%)	39 (13.1%)	17 (11.7%)	.92
Atrial fibrillation	75 (16.3%)	42 (14.1%)	24 (16.6%)	.67
Prior ischaemic stroke	93 (20.3%)	50 (16.9%)	17 (11.8%)	.061
Prior transient ischaemic attack	38 (8.3%)	16 (5.4%)	8 (5.6%)	.25
Pre-stroke mRS, median (IQR)	0 (0, 1)	0 (0, 0)	0 (0, 1)	.25
**Stroke characteristics**				
Onset to randomisation time (minutes), median (IQR)	52 (33, 103)	55.5 (34, 99)	58 (36, 88)	.95
Onset to admission time (minutes), median (IQR)	72 (52, 122)	93 (71, 135)	111 (88, 143)	<.001
Pre-hospital stroke score, median (IQR)	2 (2, 4)	2 (2, 4)	2 (2, 4)	.69
NIHSS, median (IQR)	5 (2, 11)	5 (2, 10)	5 (2, 11)	.48
Occlusion site, *n* (%)				.92
Intracranial internal carotid artery	16 (22%)	11 (25%)	4 (24%)	
Main stem of middle cerebral artery (M1)	32 (44%)	18 (41%)	10 (59%)	
Branch from middle cerebral artery (M2 or beyond)	11 (15%)	8 (18%)	2 (12%)	
Basilar artery	2 (3%)	2 (5%)	0 (0%)	
Other	8 (11%)	3 (7%)	0 (0%)	
No occlusion at the time of EVT	4 (5%)	2 (5%)	1 (6%)	
ASPECTS	7 (6, 8)	7 (6, 8)	7 (6, 8)	.76
Haematoma volume (for ICH) at admission, median mL (IQR)	11.5 (5.8, 41.9)	18.3 (5.6, 36.6)	17.7 (6.7, 33.0)	.79
Any reperfusion therapy, *n* (%)	264 (57.5%)	172 (57.7%)	85 (58.6%)	.97
Thrombectomy, *n* (%)	73 (15.9%)	44 (14.8%)	17 (11.7%)	.47
Intravenous thrombolysis, *n* (%)	232 (50.5%)	154 (51.7%)	77 (53.1%)	.86
Door-to-needle for those receiving IVT (minutes), median (IQR)	29 (25, 38)	29 (24, 36)	27 (24, 36)	.55
Onset-to-needle time for those receiving IVT (minutes), median (IQR)	104 (84, 170)	129 (103, 178)	131 (108, 162)	<.001
ICH, *n* (%)	87 (19.0%)	55 (18.5%)	23 (15.9%)	.70
Ischaemic stroke, *n* (%)	372 (81.0%)	243 (81.5%)	122 (84.1%)	
**Treatment—RIC, *n* (%)**	223 (48.6%)	149 (50.0%)	64 (44.1%)	.51
Compliance > 80%, *n* (%)	285 (63.8%)	188 (65.5%)	92 (65.7%)	.85
Compliance %, median (IQR)	88.8 (61.3, 100)	91.3 (67.5, 100)	92.5 (68.1, 100)	.93
**Stroke etiology, *n* (%)**				.80
Large artery disease	80 (21.5%)	45 (18.5%)	22 (18.0%)	
Small vessel disease	48 (12.9%)	27 (11.1%)	18 (14.8%)	
Cardioembolic	55 (14.8%)	33 (13.6%)	16 (13.1%)	
Other/rare/unknown	189 (50.8%)	138 (56.8)	66 (54.1%)	

Abbreviations: IVT = intravenous thrombolysis; RIC = remote ischaemic conditioning.

## Patients and methods

The methodology of the RESIST trial has been previously published (Clinicaltrials.gov: NCT03481777).^9^ Briefly, this was a randomised clinical trial conducted at 4 stroke centers in Denmark, that included 1500 patients with pre-hospital stroke symptoms for less than 4 hours, enrolled from 16 March 2018 to 11 November 2022, with final follow-up in 3 February 2023. Patients were randomised 1:1 to RIC or sham. The intervention was delivered using an inflatable cuff on one upper extremity (RIC cuff pressure, ≥ 200 mmHg and sham cuff pressure, 20 mmHg). Each treatment application consisted of 5 cycles of 5 minutes of cuff inflation followed by 5 minutes of cuff deflation. Treatment was started in the ambulance and repeated at least once in the hospital, and then twice daily for 7 days among a subset of participants.

The Prehospital Stroke Score assessment was performed in the ambulance and repeated 24 hours later (or at discharge, whichever occurred first) for all randomised patients.^15^ NIHSS was performed at hospital arrival and after 24 hours in the target population. At the 90-day assessment, 2 independent and blinded assessors performed a functional outcome assessment using the mRS of patients with a target diagnosis of stroke (AIS or ICH). The assessments were conducted either in person or by telephone. If a disagreement occurred, a third blinded assessor would act as the final assessor. All assessors were certified and used a validated structured mRS questionnaire.^16^ Serious adverse events qualifying as cardiovascular events during the 90-day study period were adjudicated by a blinded independent clinical event committee consisting of 3 neurologists and 1 cardiologist. Patients and outcome assessors were blinded to treatment group. Treatment adherence was registered on each device, and 80% or more received cycles of the planned number was defined as acceptable in accordance with the protocol.

### Statistical analysis plan

Our analysis was restricted to patients in the mITT population, ie, with a final diagnosis of AIS or ICH. The population was divided into 3 groups, informed by the median randomisation-to-admission time in the study (<30, 30–45, > 45 minutes). Data on baseline characteristics were summarised using frequencies and proportions for categorical variables and median with interquartile range for continuous variables. Missing data were rare and were not imputed. The primary outcome was improvement in the ordinal 90-day mRS score. Secondary outcomes included 90-day mRS 0–2 and neurological improvement according to the NIHSS at 24 hours. In addition to examining pre-hospital treatment duration-stratified outcomes in the mITT population, we additionally stratified by diagnosis (AIS, ICH) and among patients with AIS, by receipt of reperfusion therapies (no reperfusion therapies vs any reperfusion therapies, and specifically patients who received endovascular therapy, EVT). We also performed sensitivity analyses stratifying these groups by total number of RIC (or sham) cycles received prior to neuroimaging (the best achievable estimate with our data for the number of pre-hospital treatment cycles).

Ordinal logistic regression was used for the primary outcome of 90-day ordinal mRS, adjusting for age, sex, pre-hospital stroke score and treatment compliance. Binary logistic regressions, similarly adjusted, were used for the dichotomous outcome of 90-day mRS 0–2 (favourable outcome) and for the dichotomous outcome of improvement on the NIHSS by ≥ 4 points at 24 hours. Effect modification of pre-hospital treatment duration on the relationship between treatment arm and outcomes was assessed using multiplicative interaction terms in the multivariable models. All reported *P*-values were 2-sided, with *P* < .05 considered statistically significant. All analyses were performed using STATA 18.5.

## Results

Among 1500 patients who were randomised in the RESIST trial, 902 patients had a target diagnosis of stroke (737 [82%] with AIS and 165 [18%] with ICH) and constituted the mITT population. Within the mITT population, the median time spent in transport following randomisation (randomisation-to-admission time) was 29.4 minutes (IQR: 19.6–39.4 minutes). A total of 459 (50.9%) spent < 30 minutes in transport, 298 (33.0%) incurred 30–45 minutes and 145 (16.1%) incurred > 45 minutes. There were no significant differences among these 3 groups in key baseline characteristics including treatment allocation (Table 1).

**Figure 1:**
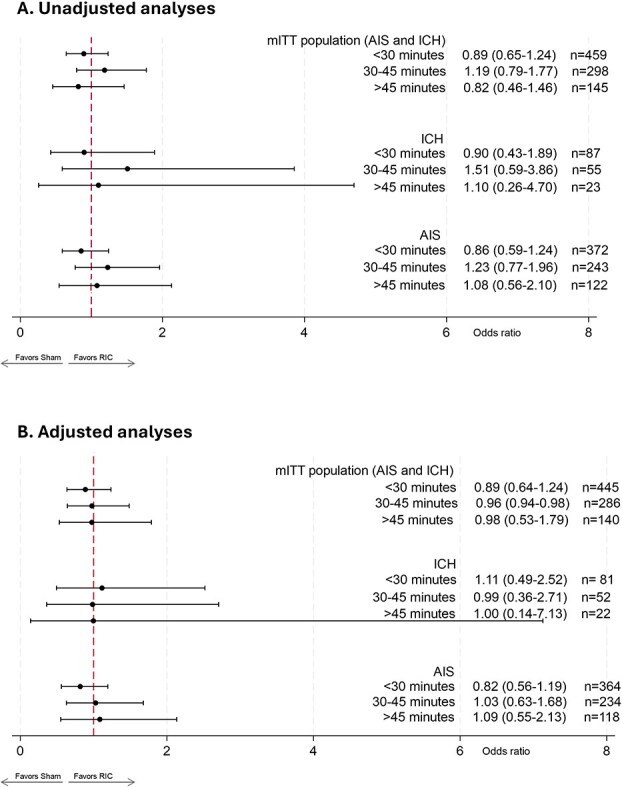
Forest plot showing (A) unadjusted and (B) adjusted effects of RIC on 90-day ordinal mRS among patients with acute ischaemic stroke and ICH, stratified by diagnosis and pre-hospital treatment time (transport time after randomisation).

There were no differences in 3-month mRS 0–2 (primary outcome), 24-hour NIHSS outcomes or mortality between the RIC and sham arms within the transport time (ie, pre-hospital treatment duration) groups (Table 2). Upon examining the effect of RIC treatment by pre-hospital treatment duration, stratified by diagnosis, there were no significant treatment effects in any of the time groups. As shown in Figure 1, both unadjusted and adjusted analyses did not show significant treatment effects of RIC on the 90-day ordinal mRS in either the combined mITT, ICH or AIS groups in any of the randomisation-to-admission time strata. There was no interaction between RIC and randomisation-to-admission time groups in relation to 90-day ordinal mRS in the mITT (*P*_interaction_ = .46), AIS (*P*_interaction_ = .48) or ICH (*P*_interaction_ = .65) populations. However, we did observe a non-significant rise in OR going from < 30 minutes to 30–45 minutes to > 45 minutes among those patients with AIS on adjusted analysis, with the effect size estimate becoming favorable (OR > 1) only for those who had > 45 minutes transport time post-randomisation (aOR: 1.09; 95% CI, 0.55–2.13). This was not observed in either the combined mITT group or the ICH group.

**Table 2:** Efficacy outcomes in patients enrolled in the RESIST trial, grouped by pre-hospital treatment duration

**Characteristic**	**Randomisation-to-admission time** **(Pre-hospital treatment duration)**								
	**<30 minutes transport** **(*n* = 459)**		***P*-value**	**30–45 min** **(*n* = 298)**		***P*-value**	**>45 min** **(*n* = 145)**		***P*-value**
	**RIC** **(*n* = 223)**	**Sham** **(*n* = 236)**		**RIC** **(*n* = 149)**	**Sham** **(*n* = 149)**		**RIC** **(*n* = 64)**	**Sham** **(*n* = 81)**	
3-month mRS 0–2, *n* (%)	152 (68.2%)	158 (66.9%)	.78	101 (67.8%)	97 (65.1%)	.62	43 (67%)	55 (68%)	.93
NIHSS at 24 hours, median (IQR)	2 (1, 7.5)	2 (1, 6)	.53	2 (0, 7.5)	3 (0, 8)	.40	2 (0, 7)	3 (1, 5)	.47
NIHSS drop ≥ 4 points	56 (26.2%)	72 (31.3%)	.23	32 (21.6%)	37 (26.1%)	.38	20 (32%)	22 (28%)	.61
NIHSS difference at 24 hours, median (IQR)	−2 (−4, 0)	−2 (−5, 0)	.22	−1 (−3, 0)	−1 (−4, 0)	.88	−2 (−4, 0)	−1 (−4, 0)	.16
Mortality, *n* (%)	17 (7.6%)	25 (10.6%)	.27	14 (9.4%)	15 (10.1%)	.85	7 (11%)	7 (9%)	.64
Quality of life, WHO-5 Well-Being Index, median (IQR)	72 (60, 84)	76 (64, 88)	.23	76 (60, 84)	72 (64, 88)	.94	72 (68, 84)	78 (64, 86)	.82

**Figure 2:**
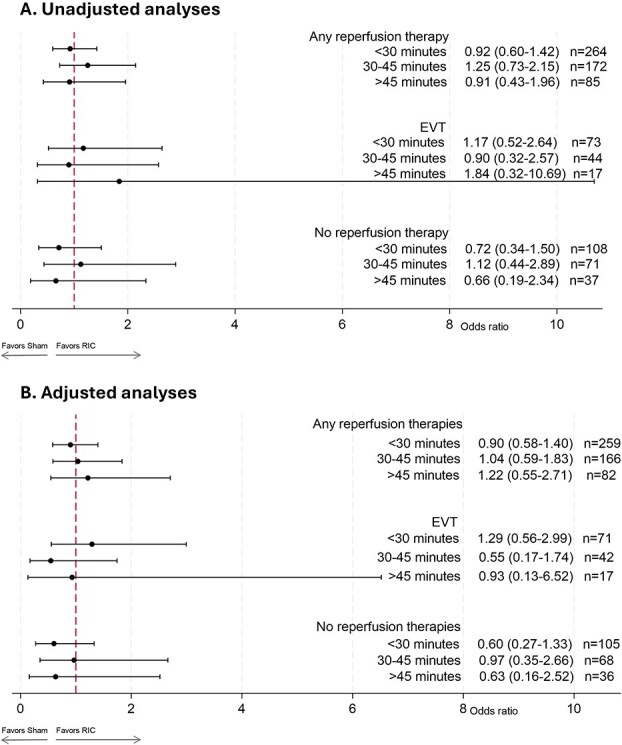
Forest plot showing (A) unadjusted and (B) adjusted effects of RIC on 90-day ordinal mRS among patients with acute ischaemic stroke, stratified by receipt of reperfusion therapies and transport time after randomisation.

Upon further stratifying patients with AIS by randomisation-to-admission time and based on receipt of reperfusion therapies, we found no significant differences across time. As shown in Figure 2, both unadjusted and adjusted analyses did not show significant treatment effects of RIC on the 90-day ordinal mRS in patients with AIS who received any reperfusion therapy, in those who received EVT or in those who received no reperfusion therapies, in any of the randomisation-to-admission time strata. There was no interaction between RIC and randomisation-to-admission time groups in relation to 90-day ordinal mRS among those patients who received any reperfusion therapy (*P*_interaction_ = .59), including the subset who received EVT (*P*_interaction_ = .74) or those who did not receive any reperfusion therapies (*P*_interaction_ = .84). However, on adjusted analyses, the highest effect size estimate in favour of treatment, although still non-significant, was seen only for patients receiving reperfusion therapies who had > 45 minutes transport time post-randomisation (aOR: 1.22; 95% CI, 0.55–2.71).

Sensitivity analyses stratifying the mITT, ICH and AIS groups (Figure SS1) and the reperfusion therapy groups (Figure SS2) by the total number of RIC cycles received prior to neuroimaging yielded similar results, with no significant effects or differences seen across any of the strata.

**Figure 3:**
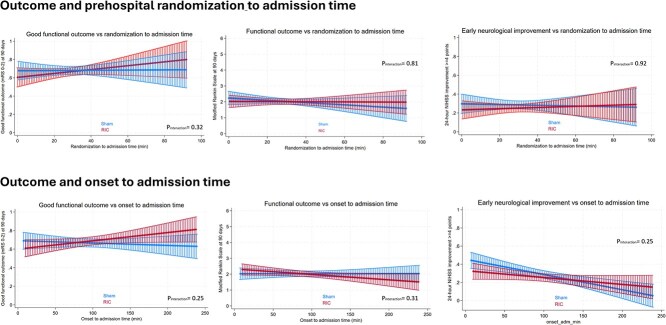
Association between timing metrics and outcomes in patients with acute ischaemic stroke randomised to remote ischaemic conditioning (RIC) or sham. Each panel displays a fitted probability curve with 95% confidence intervals for outcomes across time from randomisation or symptom onset to hospital admission. Sham and RIC groups are marked in the figure. Top row: Time from randomisation to admission versus (left) probability of good functional outcome (mRS 0–2), (middle) median mRS score at 90 days, (right) proportion with early neurological improvement (≥4-point NIHSS reduction at 24 hours). Bottom row: Time from symptom onset to admission versus the same 3 outcomes. Curves are estimated using linear prediction with 95% confidence intervals (capped). Remote ischaemic conditioning and sham groups are analysed separately.

When examining other outcomes of interest, in the AIS population, there was no interaction between RIC and randomisation-to-admission time groups in relation to 90-day mRS 0–2 (*P*_interaction_ = .99), or early NIHSS improvement (*P*_interaction_ = .48). Similarly, there was no interaction between RIC and onset-to-admission time in relation to ordinal mRS (*P*_interaction_ = .65), mRS 0–2 (*P*_interaction_ = .75) or NIHSS improvement (*P*_interaction_ = .61). Findings were similar when plotting outcomes in the RIC and sham groups against randomisation-to-admission or onset-to-admission times when the latter were modeled as continuous variables. As shown in Figure 3, there were no significant differences between the RIC and sham groups in ordinal mRS scores, attainment of good functional outcome (mRS 0–2) or attainment of early NIHSS improvement across the spectrum of randomisation-to-admission times, with similar findings seen when considering onset-to-admission times. A notable exception was that the odds of better outcomes appeared to improve with longer times for the RIC group in contrast to the decline over time seen in the sham group, especially for good functional outcome (90-day mRS 0–2). However, no significant interactions were observed between treatment assignment and either randomisation-to-admission time or onset-to-admission time.

As for the ICH population, there was no interaction between RIC and randomisation-to-admission time in relation to 90-day mRS 0–2 (*P*_interaction_ = .61), or early NIHSS improvement (*P*_interaction_ = .44). There was also no interaction between RIC and onset-to-admission time in relation to ordinal mRS (*P*_interaction_ = .18), mRS 0–2 (*P*_interaction_ = .44) or NIHSS improvement (*P*_interaction_ = .34).

## Discussion

In this post-hoc analysis of the RESIST randomised-controlled trial, we found that there were no significant treatment effects of RIC compared to sham in either the combined mITT, ICH or AIS groups when stratified by pre-hospital treatment duration (randomisation-to-admission time). Further stratifying patients with AIS based on receipt of reperfusion therapies also revealed no significant differences in RIC efficacy across pre-hospital treatment duration. There was no interaction between RIC and pre-hospital treatment duration groups in relation to 90-day ordinal mRS, mRS 0–2 or early NIHSS improvement in the AIS population; findings were similar in the ICH population and when examining onset-to-admission times.

Studies of RIC in acute stroke have had conflicting findings. In particular, in contrast to RESIST, The Remote Ischemic Conditioning for Acute Moderate Ischemic Stroke (RICAMIS), a large randomised-controlled trial of RIC in 1776 patients in China with AIS, found that RIC was associated with significantly better rates of excellent functional outcome (90-day mRS score of 0–1), 67.4% versus 62.0%.^17^ However, RIC was not initiated in the pre-hospital setting in RICAMIS and there were also concerns regarding blinding. In examining pre-hospital treatment duration in more detail, we sought to understand whether differences in exposure to the therapy—which seem relevant for other promising neuroprotective therapies like nerinetide—may help differentiate patients who benefit from RIC, particularly among patients with AIS who went on to receive reperfusion therapies.^13^^,^^18^ We found no significant findings in this regard, indicating that heterogeneity in pre-hospital treatment duration probably did not contribute to the neutral results of the RESIST trial.

That being said, we did observe non-significant trends towards increasing odds of functional improvement with RIC versus sham in patients with AIS, including in the subset who received reperfusion therapies, upon stratifying by pre-hospital treatment duration. There were no significant interactions between treatment assignment and time. There was an unexpected, although non-significant, observation of improved rates of good functional outcome in the RIC group with increasing pre-hospital times (randomisation-to-admission), in contrast to the expected decline in good outcomes with increasing pre-hospital delays that is expected in typical acute stroke, as was observed in the sham group.^19–21^ This potentially spurious finding should be cautiously interpreted and it is unclear how these observations may extrapolate over longer durations or a larger sample.

In the broader context of considering the overall landscape of RIC in hyperacute stroke care, our results imply that future pre-hospital trials of RIC are unlikely to show benefit when conducted under similar treatment durations. In this regard, pre-hospital transport/treatment times in the RESIST trial were relatively brief, with nearly all the patients spending under an hour in transport, limiting our ability to robustly examine the association of longer exposures to RIC prior to reperfusion or other definitive stroke therapies. Therefore, future studies of RIC could examine the influence of pre-hospital treatment duration in regions where patients with large vessel occlusion strokes are routinely transported for several hours prior to receiving reperfusion therapies or other hospital-based stroke care.^3^^,^^22^

The study had relevant additional limitations. The findings are based on subgroup analyses within the RESIST randomised-controlled trial which was not specifically powered to study differences in RIC outcome by pre-hospital treatment duration, and therefore, our results should be interpreted cautiously. In particular, our neutral results may reflect type II error from inadequate power for interaction effects. We used randomisation-to-admission time as a proxy for pre-hospital RIC treatment time, but patients may have experienced variable delays from randomisation to treatment initiation. Although personnel were instructed to initiate the treatment right away following randomisation, we did not have the exact data for time from randomisation to RIC treatment initiation. We were also unable to adjust for potential radiological factors such as extent of early ischaemic change or haemorrhage, occlusion location or brain frailty, that could influence post-stroke outcomes.^23^ In the subset with AIS, stroke etiology was not considered in this analysis, which might influence the response to RIC according to some post-hoc analyses of the RICAMIS and RESIST trials.^24^ However, these have had divergent conclusions, with large artery atherosclerotic etiologies being associated with positive outcomes in RICAMIS versus small vessel disease in RESIST.^25^^,^^26^

In conclusion, longer pre-hospital treatment duration was not associated with efficacy of RIC in patients with AIS or ICH in the RESIST trial including among patients with AIS who received reperfusion therapies, although the durations were generally under an hour. Our findings may not apply to rural or resource-limited settings where RIC could be routinely administered over longer periods. Overall, these results show no treatment duration-dependent benefit of RIC in acute stroke, at least in settings where patients are transported quickly.

## Data sharing

The data that support the findings of this study are available from the corresponding author upon reasonable request. Individual participant data that underlie results in this article will be shared after deidentification. Proposals should be directed at rolfblau@rm.dk. To gain access, data requestors will need to sign a data processing agreement.
